# Implantation of Mouse Embryonic Stem Cell-Derived Cardiac Progenitor Cells Preserves Function of Infarcted Murine Hearts

**DOI:** 10.1371/journal.pone.0011536

**Published:** 2010-07-12

**Authors:** Nicolas Christoforou, Behzad N. Oskouei, Paul Esteso, Christine M. Hill, Jeffrey M. Zimmet, Weining Bian, Nenad Bursac, Kam W. Leong, Joshua M. Hare, John D. Gearhart

**Affiliations:** 1 Department of Biomedical Engineering, Pratt School of Engineering, Duke University, Durham, North Carolina, United States of America; 2 Miller School of Medicine, University of Miami, Miami, Florida, United States of America; 3 Institute for Regenerative Medicine, University of Pennsylvania, Philadelphia, Pennsylvania, United States of America; 4 Interventional Cardiology, University of California San Francisco, San Francisco, California, United States of America; University of Cincinnati, United States of America

## Abstract

Stem cell transplantation holds great promise for the treatment of myocardial infarction injury. We recently described the embryonic stem cell-derived cardiac progenitor cells (CPCs) capable of differentiating into cardiomyocytes, vascular endothelium, and smooth muscle. In this study, we hypothesized that transplanted CPCs will preserve function of the infarcted heart by participating in both muscle replacement and neovascularization. Differentiated CPCs formed functional electromechanical junctions with cardiomyocytes *in vitro* and conducted action potentials over cm-scale distances. When transplanted into infarcted mouse hearts, CPCs engrafted long-term in the infarct zone and surrounding myocardium without causing teratomas or arrhythmias. The grafted cells differentiated into cross-striated cardiomyocytes forming gap junctions with the host cells, while also contributing to neovascularization. Serial echocardiography and pressure-volume catheterization demonstrated attenuated ventricular dilatation and preserved left ventricular fractional shortening, systolic and diastolic function. Our results demonstrate that CPCs can engraft, differentiate, and preserve the functional output of the infarcted heart.

## Introduction

Cardiomyocyte loss as a result of myocardial infarction (MI) injury is considered irreversible with the heart lacking sufficient capacity for self-regeneration. Cell-based cardiac therapies are proposed as an attractive therapeutic alternative to reverse cardiomyocyte loss, repair the injured myocardium and ultimately prevent heart failure. To date, a variety of cell sources, both of adult and embryonic origin, have been investigated for use in heart repair with mixed outcomes [1], [2]. The use of adult cells is attractive because of their immunocompatible nature, ease of isolation, restricted differentiation potential, and capacity to proliferate rapidly. However, inadequate potential for cardiac differentiation or integration with host cells, limits the benefit of these cells mainly to their paracrine action. On the other hand, embryonic stem cells (ESCs) are able to differentiate into relatively large numbers of early stage cardiomyocytes that functionally integrate with host heart cells [3], [4], [5]. While ESC-derived cardiomyocytes have been successfully applied for the treatment of myocardial infarction in animal models [6], [7], [8], [9], their clinical application is currently hampered by their neoplastic and immunogenic potential [10].

We and others have recently described the identification, isolation, and characterization of the novel mouse ESC-derived cardiac progenitor cells (CPCs) on the basis of *Brachyury*/*Flk1* [11], *Isl1/Flk1/Nkx2-5* [12], *cKit*/*Nkx2-5* [13], or *Nkx2-5* [14] expression. These cells represent a promising source for heart repair as they have the restricted capacity to differentiate into cardiac muscle, smooth muscle, and vascular endothelium [11], [12], [13], [14]. In this study we hypothesized that mouse ESC-derived CPCs will exert functional improvement after myocardial infarction primarily through their multipotential differentiation capacity as well as through the formation of stable and integrated grafts within the host myocardium. We found that when co-cultured with neonatal rat ventricular cardiomyocytes (NRMVs), the CPCs differentiated into cardiomyocytes, formed gap junctions with the rat cells, and supported electrical propagation over a centimeter-scale distance. Temporal assessment performed as long as one month after injection into the infarcted region of the murine myocardium, demonstrated that the CPCs engrafted and differentiated into cardiomyocytes, as well as contributed to neovascularization in the infarcted region. The differentiated cardiomyocytes also formed gap junctions with the host myocardium. The animals that received the CPCs demonstrated significantly improved cardiac function as assessed by echocardiography and pressure/volume (PV) loop analysis. No teratoma formation was observed following cell transplantation.

## Results

### Isolation and *in vitro* characterization of mouse ESC-derived CPCs

**Figure 1:**
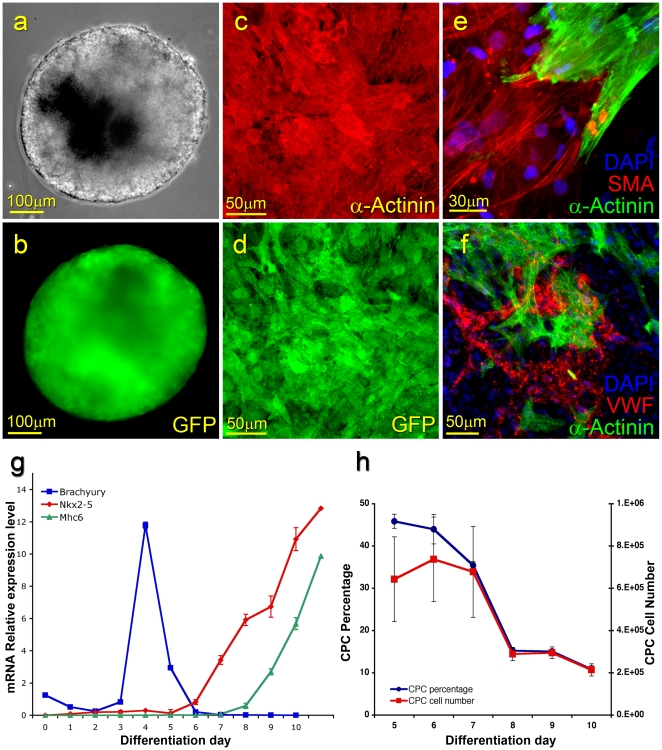
Derivation and *in vitro* characterization of mouse ESC-derived CPCs. ESCs were induced to differentiate through 3D aggregation and embryoid body formation. Clusters of CPCs, located within the embryoid body, expressed GFP under the control of the Nkx2-5 enhancer element (a–b). GFP^(+)^ CPCs isolated by FACS sorting differentiated exclusively into cardiomyocytes (α-Actinin) while retaining GFP expression (c–d). The CPCs differentiated *in vitro* into cardiomyocytes (α-Actinin), smooth muscle (Smooth muscle actin), and endothelial cells (Von Willebrand factor) (e–f). Temporal quantitative RT-PCR analysis for the nascent mesoderm marker *Brachyury*, the cardiac progenitor marker *Nkx2-5*, and a cardiomyocyte marker *Mhc6* (g). The percentage of GFP^(+)^ CPCs was determined temporally following induction of mouse ESC differentiation (h).

The mouse ESC lines *D3* [15] and *Rosa26* [16] were stably transfected with DNA constructs allowing the expression of the green fluorescent protein (GFP) under the control of the mouse cardiac specific enhancer element of the Nkx2-5 transcription factor as previously described [14]. Following isolation of 50 colonies (clonal) for each cell line, stably transfected clones were identified and further used based on their capacity to express GFP selectively in the spontaneously contracting cardiomyocyte cell clusters. Mouse ESCs were induced to differentiate in suspension forming aggregates termed embryoid bodies (EBs) and initial detection of GFP coincided with initiation of *Nkx2-5* expression on differentiation day 5 (**Figs. 1a, b**).

Temporal quantitative RT-PCR analysis performed on differentiating ESCs indicated a time period (days 5–6) during which the CPCs were present but not yet committed into specific cell lineages (**Fig. 1g**). Prior to initiation of *Nkx2-5* expression, coinciding with cardiac progenitor induction, the detection of *Brachyury* transcripts (day 4) indicated the formation of nascent mesoderm. By day 7, the CPCs underwent differentiation-commitment into cardiac cell lineages including cardiomyocytes as indicated by the initiation of *Mhc6* expression along with the detection of spontaneously contracting cell clusters.

The initial number and percentage of GFP^(+)^ CPCs decreased temporally between days 5 and 10 (**Fig. 1h**). This observation, which has been previously reported [13], may be a result of proliferation of other cell types, epigenetic silencing of the specific *Nkx2-5* enhancer element used in this set of experiments, and/or differentiation towards the vascular and smooth muscle endothelium cell lineages which downregulate *Nkx2-5*.

To assay the differentiation capacity of the CPCs, following isolation of the GFP^(+)^ progenitor population, the cells were reaggregated and the cell clusters were induced to differentiate for 7–30 days. Several foci of GFP^(+)^ spontaneously contracting cardiomyocytes (α-Actinin expression) were detected in culture (**Figs. 1c, d**). The multi differentiation potential of the CPCs was determined by demonstrating cell specific expression of cardiac α-Actinin, endothelial Von-Willebrand factor, and smooth muscle actin in the differentiating clusters (**Figs. 1e, f**). This was also confirmed by the RT-PCR analysis (previously demonstrated [14]). No colonies of undifferentiated ESCs were detected as confirmed by the absence of *Pou5f1* and *Nanog* expression.

### Determination of the electrocoupling capacity of CPCs

**Figure 2:**
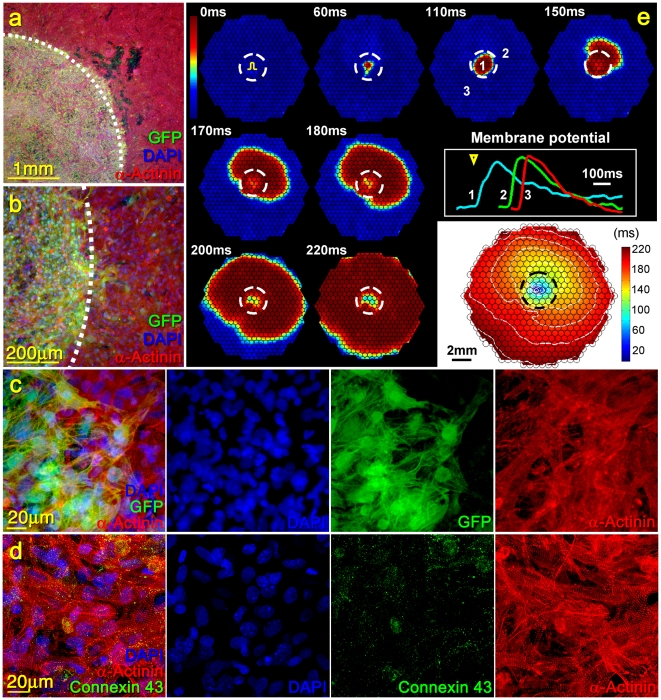
Structural and functional assessment of a NRVM monolayer with a central island comprised of differentiated CPCs. A GFP^(+)^ CPC island (green) within the cardiac network (red) (a–b). Composite image and separate fluorescence channels showing the border between NRVMs and CPCs (c). Note that significant number of GFP positive cells are also α-Actinin positive (cardiomyocytes). Composite image and separate channels showing the Connexin 43 staining within the CPC island. While NRVMs were connected via relatively long Connexin 43^(+)^ gap-junction plaques (data not shown), the gap-junctions in CPC differentiated cardiomyocytes appear small and irregular (d). Electrical propagation initiated inside the CPC island propagated into the surrounding cardiac network (e). Individual hexagonal frames denote 19.5 mm diameter recording area with membrane voltage snapshots shown at given times. Electrical stimulus (pulse sign) was applied in the center of the CPC island at time 0 ms. Membrane voltage is color coded from rest (blue) to peak (red). Circles denote 504 recordings sites. Dashed white line denotes the CPC island. Membrane potentials at selected sites within (1) and outside (2&3) the CPC island are also shown. Electrical stimulus (yellow triangle) yielded action potential propagation that was significantly slower within the island than in the surrounding cardiomyocytes. Isochrones of electrical propagation (white lines) are shown at the bottom right of. Note central isochrone crowding due to slow propagation within the CPC island (black dashed line).

The potential of CPC derived cardiomyocytes to form functional gap junctions was determined by utilizing a previously described *in vitro* NRVM co-culture assay [17], [18] which allows the observation of action potential propagation by the means of optical mapping over a macroscopic (3 cm2) culture area [18]. In particular, the CPCs were selectively seeded at high density in the central region of pre-masked coverslips with the NRVMs surrounding them (**Fig. 2a**). The co-cultures exhibited distinct border between the CPCs and the NRVMs as assayed by GFP and α-Actinin expression (**Figs. 2a, b**). Immunostaining for cardiomyocyte, smooth muscle and endothelial markers after 14 days of co-culture demonstrated that the majority of the CPCs differentiated into cardiomyocytes (**Fig. 2c**) with limited smooth muscle or endothelial cell differentiation (data not shown). On the other hand, CPCs plated at low density in the central region of the coverslips formed few patches of cardiomyocytes with the majority of cells differentiating into smooth muscle cells (**Fig. S1**). Gap junctions indicated by punctuate Connexin 43 localization at the site of cell-cell contacts were detected in the differentiated cardiomyocytes (**Fig. 2d**). However, unlike the NRVMs that expressed Connexin 43 in relatively long plaques at the sites of cell contacts, the CPC-derived cardiomyocytes expressed Connexin 43 sporadically and irregularly.

Optical mapping in all co-cultures revealed spontaneous electrical activity at a relatively low rate (1.2±0.3 Hz) that originated in the CPC islands, suggesting the existence of pacemaking cells amongst the CPC-differentiated cardiomyocytes. Consistent with positive staining for Connexin 43, both spontaneous and pacing-induced action potentials (2 Hz rate) within the CPC island propagated outwards into the surrounding NRVM area (**Fig. 2e**). Action potentials also propagated into the CPC island when initiated from the surrounding NRVM area (data not shown). The apparent velocity of electrical propagation inside the CPC island was significantly lower as compared to that of the surrounding NRVMs (1.6±0.2 vs. 16.7±1.5 cm/s) and dependent on the efficiency of CPC differentiation into cardiomyocytes. Specifically, lower numbers of sparsely distributed cardiomyocytes within the central island yielded significantly lower velocity of propagation (0.4±0.1 cm/s, **Fig. S1g**).

### CPC injection in the mouse infracted myocardium

**Figure 3:**
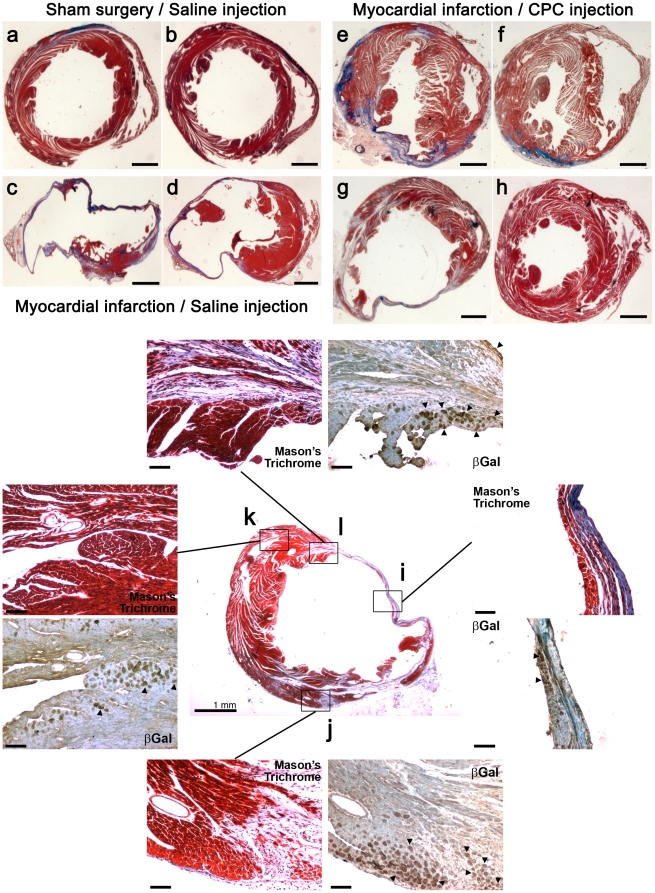
Scar tissue formation and CPC detection. Histological analysis (Mason's trichrome staining) and immunohistochemical analysis of the infarcted myocardium demonstrating the extent of scarring (blue) in the myocardium of the three experimental groups at two parallel cross-sections of the myocardium. Sham surgery and saline injections (a–b). Myocardial infarction and saline injections (c–d). Myocardial infarction and CPC injections (e–h). Scale bar: 1 mm**.** High magnification panel pairs show parallel thin cross-sections stained either with Mason's trichrome stain or with an antibody against the β-Galactosidase protein which is constitutively expressed in the injected cells (Rosa 26 mouse ESC line). Scar region (i), Interface between the scar and healthy myocardium (j–l). Healthy myocardium (k). Black arrowheads denote β-Galactosidase^(+)^ cells within the myocardium.

The potential of CPCs to treat myocardial infarction injury was assessed by evaluating their ability to form stable grafts in the host myocardium, differentiate into lineage-committed cells, form *in vivo* gap junctions, and ultimately improve the functional output of the injured hearts. To determine the extent of injury and scar tissue formation in the infarcted hearts, we performed Mason's trichrome staining on thin heart sections that allowed us to distinguish healthy muscle tissue (red) from scar tissue (blue) (**Fig. 3**). The control group that underwent sham surgery displayed only minor scarring which could be attributed to injury sustained during the saline injections (**Figs. 3a, b**). The hearts that underwent LAD ligation and received only saline injections sustained massive infarcts that encompassed almost the entire left ventricle (including apex) and exhibited thin scarred walls and extensive remodeling (**Figs. 3c, d**). In contrast, the animals that underwent LAD ligations and received CPCs displayed smaller zones of scar tissue formation with decreased cardiac remodeling (**Figs 3e–h**). While a few hearts displayed minor tissue scarring (**Figs. 3e, f**) similar to that observed in the sham hearts, others displayed a certain degree of wall thinning and remodeling (**Figs. 3g, h**). In this case, however, the scar was localized only around the ligation site.

To assess the engraftment potential and distribution of the transplanted cells irrespective of their lineage differentiation phenotype within the myocardium we compared parallel serial tissue cross-sections pre-treated either with Mason's trichrome stain or anti *β-Galactosidase*, which is constitutively expressed in the Rosa26-derived CPCs (**Figs. 3i, l**). *β-Gal* ^(+)^ cells were detected within the infarcted region in an area comprising a thin muscle band underneath the scar tissue (**Fig. 3i**), borders between the scar tissue and healthy muscle (**Figs. 3j, k**), as well as within the healthy muscle in the vicinity (**Fig. 3l**) but not remote (not shown) from the scar. These results suggested that the transplanted cells successfully engrafted at all three injection areas including the center of the infarct.

Anti *β-Galactosidase*-specific staining was also used in order to determine the survival rate of the transplanted cells one month post intramyocardial delivery. *βGal* ^(+)^ cells was detected and summed in multiple sections both within and in the surrounding area of the myocardial infarction region. The survival rate was calculated by extrapolating the number of *βGal* ^(+)^ cells over the entire estimated area of the affected myocardium (≈4 mm) and determined to be 12.7±2.1% of the initial total number of cells injected.

**Figure 4:**
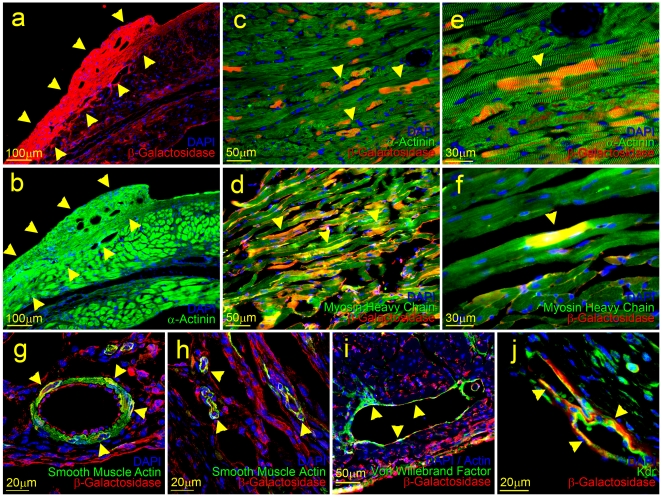
Differentiation potential of CPCs in the infarcted myocardium. Low magnification images demonstrating large-scale engraftment and differentiation of the injected CPCs into the cardiomyocyte cell lineage (a–b). Panels (a) and (b) show parallel thin sections of the infarcted myocardium. Yellow arrowheads denote the newly formed β-Gal^(+)^ and α-Actinin^(+)^ myocardium. High magnification images demonstrating that the transplanted CPCs primarily differentiated into cardiomyocytes (c–f). Large and small newly formed vessels located within the infarcted region of the myocardium contain a combination of host and donor cells. Yellow arrowheads denote the β-Gal^(+)^ donor cells expressing smooth muscle actin (g, h), Von Willebrand factor (i), and KDR (j).

We proceeded to examine the differentiation commitment of the transplanted cells. Large tissue areas surrounding the injury site co-stained for *β-Galactosidase* and *α-Actinin* (**Figs. 4a, b**) indicating robust differentiation of the transplanted cells into cardiomyocytes. At higher magnification, a large number of individual or clustered CPC-derived cardiomyocytes were detected incorporated within the host cardiac cell network (**Figs. 4c–f**). While the presence of rod cell shape and cross-striations (**Fig. 4e**) indicated advanced cell differentiation, smaller size of CPC-derived cardiomyocytes compared to host cardiomyocytes suggested maturation level inferior to that of the adult cells. The majority of the *β-Gal* staining also co-localized with another cardiac muscle marker *Myosin Heavy Chain* (*MHC*, **Figs. 4d, f**).

In the infarcted region of animals which received CPC injections several blood vessels comprised of both donor and host cells were detected (**Figs. 4g–j**). The *βGal* ^(+)^ CPCs readily differentiated into vascular smooth muscle and incorporated in large and small vessels with *βGal* ^(-)^ host cells (**Figs. 4g, h**). The CPCs also differentiated into endothelial cells in some of the detected blood vessels as determined by the detection of *Vwf* and *Kdr* expression (**Figs. 4i, j**). Small *βGal* ^(+)^ uncommitted progenitors were also detected. Taken together, ESC-derived CPCs committed predominantly to the cardiac muscle cell fate both when transplanted in the infarcted heart and when densely seeded in co-culture with rat cardiomyocytes in addition to inducing vascularization.

Since cardiomyocytes in the adult mouse myocardium are binucleated whereas embryonic stem cell-derived cardiomyocytes are immature and primarily uninucleated [19] we were able to delineate differentiation versus cell fusion events by determining the number of *βGal* ^(+)^ cardiomyocytes containing a single nucleus. In our analysis approximately 96% of the identified cells contained a single nucleus indicating that at least that number of cells were the result of direct differentiation versus cell fusion.

### Electrical and mechanical coupling assessment of CPCs

**Figure 5:**
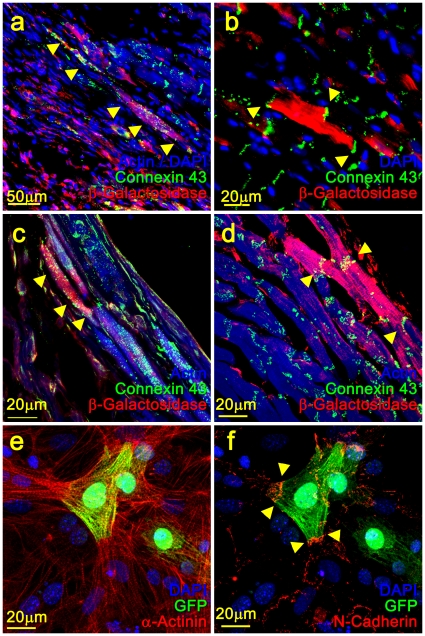
Electromechanical coupling capacity of CPCs. Electrical and mechanical coupling of differentiated CPCs with the host myocardium and neonatal rat ventricular myocytes. β-Galactosidase^(+)^ cardiomyocytes form gap junctions as demonstrated by Connexin 43 immunostaining within the infarcted region (a, c), as well as in the healthy myocardium (b), and at the border area between healthy and infarcted myocardium (d). CPCs differentiate *in vitro* into GFP^(+)^/α-Actinin^(+)^ cardiomyocytes (e) and mechanically couple with co-cultured neonatal rat ventricular myocytes as determined by N-Cadherin detection at the border region between the two cell types (f).

The capacity of the differentiated CPCs to electrocouple *in vivo* with the host myocardium was examined by immunohistochemical analysis (**Figs. 5a–d**). The majority of the *βGal* ^(+)^ cardiomyocytes which also exhibited Connexin 43 gap-junction staining were detected at the site of the infarct (**Figs. 5a, c**). A small number of the donor cells were detected within the healthy myocardium (**Fig. 5b**) or at the border between healthy tissue and infarct (**Fig. 5d**).

The capacity of the differentiated CPCs to form mechanical inter-connections with neonatal rat ventricular cardiomyocytes was assessed *in vitro* (**Figs. 5e, f**). Isolated GFP^(+)^ CPCs were co-cultured with NRVMs and subsequently assayed for GFP and *N-Cadherin* expression which mediates adhesion in the intercalated discs at the termini of cardiomyocytes thereby serving as a mechanical anchor for myofibrils at cell-cell contacts [20]. The GFP^(+)^ cardiomyocytes, which were readily distinguishable from the NRVMs, formed *N-Cadherin* ^(+)^ intercalated disc cell contracts with the NRVMs.

### Determination of cardiac functional output by echocardiographic evaluation

**Figure 6:**
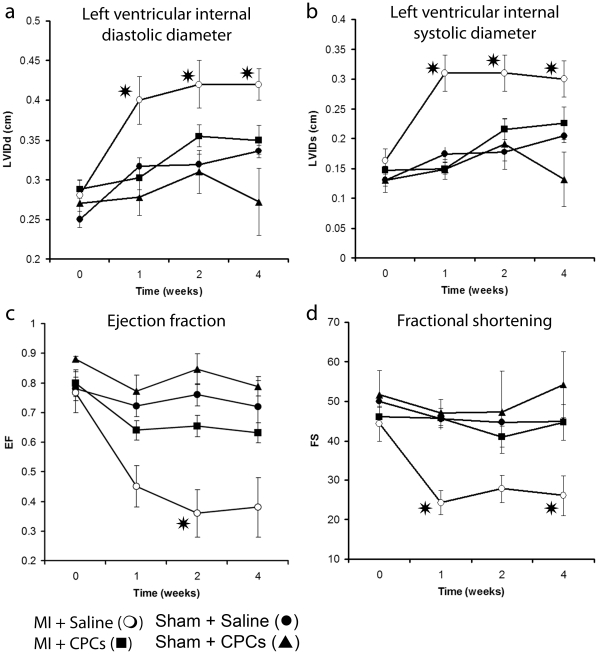
Echocardiographic analysis. Time course of structural and hemodynamic indices obtained using echocardiography in the four studied groups. The left ventricular size in diastole (a) and systole (b) increased in the saline injected infarct control group while in CPC treated and sham groups it remained stable over 4 weeks after MI injury (p<0.01). Similarly, the ejection fraction (c) and fractional shortening (d) decreased in the saline infracted control group while in CPC treated groups remained stable over 4 weeks post infraction (p<0.01). Note that over the course of treatment neither of 4 indices differed between CPC treated infracted heart and sham groups injected with saline or CPC.

Myocardial infarction injury produced a time-dependent ventricular dilatation and left ventricular dysfunction (**Fig. 6**). Over a 4-week time span the control group (saline-injected infracted animals), exhibited a 50% increase (2.80±0.2 mm to 4.2±0.2 mm) of the left ventricular internal diastolic diameter (LVIDd, **Fig. 6a**) and an 84% increase (1.63±0.2 mm to 3.0±0.3 mm) of the left ventricular internal systolic diameter (LVIDs, **Fig. 6b**). Transplantation of CPCs significantly attenuated the cardiac remodeling, yielding a smaller, 21%, increase of the LVIDd (2.88±0.1 mm to 3.5±0.18 mm) and a 53% increase of the LVIDs (1.47±0.07 mm to 2.26±0.27 mm).

Over the span of 4 weeks the ejection fraction (EF, **Fig. 6c**) in the control group (saline-injected infracted animals) decreased from 76.8±6.9% to 38.0±10%, while for the CPC-treated infracted animal group there was a lesser ejection fraction decrease (79.9±4% to 63.1±3%) which was also comparable to that of the sham groups, thus, indicating preserved hemodynamic function. Consistent with these results, fractional shortening (FS) following infarction injury and saline injection was significantly reduced from 44.29±4.4% before LAD ligation to 26.08±5.07% after 4 weeks. In contrast, CPC injection in the infracted zone preserved the FS (46.04±2.32% to 44.66±4.5%) at levels measured prior to the infarction injury, and similar to the results obtained for the sham groups (**Fig. 6d**).

#### Survival

A total of 55 surgical procedures were performed with a surgical mortality of 10.9% (**Fig. S2**). Forty mice finished the 4 weeks of the study, of which 26 underwent successful hemodynamic analysis with a pressure-volume catheter.

### Determination of cardiac functional output by pressure-volume analysis

**Figure 7:**
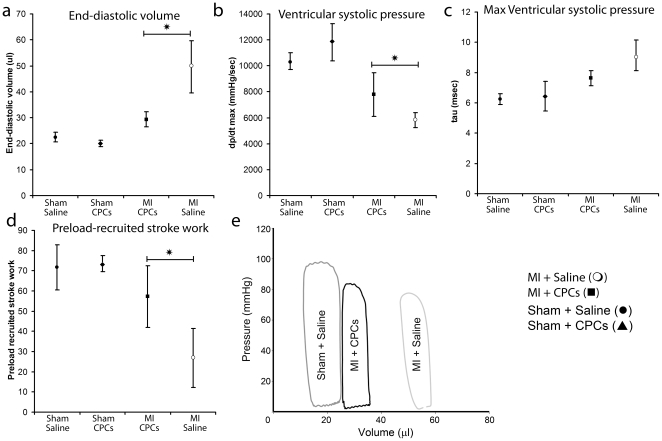
Pressure-Volume loop analysis. Left ventricular pressure-volume analysis in the four groups 4 weeks post infarction. The CPC treated group exhibited preservation of ventricular volume in diastole (a) (p<0.01). An improved relaxation constant (Tau-Weiss) is observed in CPC treated mice (c) (p<0.01). (dP/dt)_max_ (b) and preload recruitable stroke work (PRSW) (d) was improved in the CPC treated group exhibiting preserved contractility parameters. (e) Sample Pressure-volume loop recordings from individual mice four weeks after myocardial infarction. While the sham treated mouse has kept its end-diastolic and end-systolic volumes these parameters have increased in MI ones with less maximum pressure, however the degree of dilatation and remodeling seems increased in the saline treated mouse as compared to the one receiving CPCs.

In order to further access the cardiac functional capacity of the four animal groups we performed Pressure-Volume loop analysis (**Fig. 7**) [21]. Consistent with the echocardiographic analysis of ventricular dilatation, the end diastolic volume (EDV, **Fig. 7a**) for the saline-injected infracted animals measured 49.5±10.0 µl, as compared to 29.4±2.9 µl for the CPC-treated infracted animal group. The animals that underwent sham surgery and received either cells or saline injections measured 20.1±1.2 µl and 22.5±1.9 µl respectively.

Hemodynamic catheterization also allowed the measurement of the indices of myocardial contractility (**Fig. 7b**). Peak pressure rise ((dP/dt)_max_) is a measure of the fastest pressure rise within the left ventricle during the isovolumetric phase of myocardial contraction and it can be correlated with the peak acceleration of blood in the descending aorta through the ventricle. The left ventricular peak pressure rise ((dP/dt)_max_) for the control group (saline-injected infracted animals, 5830.3±584.5 mmHg/sec) was lower than that of the CPC-treated infracted animals (8427.3±1850.8 mmHg/sec), which in turn was comparable to the ((dP/dt)_max_) values measured for the saline-treated sham animals (10,373.4±649.5 mmHg/sec) but lower than those measured for the cell-treated sham animals (11,826.0±1433.6 mmHg/sec).

The measurement of the relaxation time constant (**Fig. 7c**), which measures the speed of isovolumic relaxation during early diastole, revealed that the diastolic relaxation of the CPC-treated infarcted group was faster (7.6±0.5 msec) than that of the saline-treated infarcted group (9.1±1.0 msec) and comparable to the sham surgery control groups (6.2±0.3 msec for saline-treated and 6.5±1.0 msec for CPC-treated). Taken together, the treatment of infracted hearts with transplanted CPCs resulted in a significant improvement of both systolic and diastolic indices of cardiac function compared to untreated, saline injected controls (to levels comparable to those found in uninfarcted animals).

The measurement of *preload recruitable stroke work* (PRSW) relationship, which describes the dependence of the stroke work on the end diastolic volume, demonstrated a preservation of the cardiac functional capacity in the cell-treated infarcted group (**Fig. 7d**). Specifically, the saline-treated group exhibited significantly lower PRSW slope (26.8±14.6 mmHg) compared to cell-treated group (57.3±15.3 mmHg), which in turn remained comparable to both sham groups (**Fig. 7d**).

## Discussion

The main aim of this study was the determination of the therapeutic capacity of the recently described ESC-derived cardiac progenitor cells [14] to treat myocardial infarction injury. We first describe the *in vitro* differentiation potential of mouse ESC-derived CPCs. We further demonstrate the capacity of the CPC-derived cardiomyocytes to form functional gap junctions, propagate action potentials over a relatively large area, and electrically and mechanically connect with primary ventricular cardiomyocytes in culture. Finally, we demonstrate a stable engraftment and robust cardiomyocyte differentiation of the transplanted CPCs following myocardial infarction injury, formation of gap junctions with host cells, as well as significant preservation of cardiac function in the CPC-treated animals compared to the untreated group.

A significant aspect of the study is the utilization of a novel cell progenitor: ESC-derived CPCs selected based on the activity of a cardiac specific *Nkx2-5* [22] enhancer. We recently reported that the CPCs closely recapitulate the earliest stages of *in vivo* cardiac development and are capable of multipotential differentiation into cell types that contribute to the heart [14]. Success of cell-based cardiac therapies is dependent not only on the ability of transplanted cells to differentiate into cardiomyocytes and functionally integrate with host cells, but also on the adequate vascularization of the newly formed tissue. The cardiovascular differentiation potential of the *Nkx2-5^(+)^* CPCs and the relative ease to obtain large cell numbers represent major advantages for their potential use as a single therapeutic cell source. Importantly, since these CPCs are capable of generating both cardiomyocytes and vascular cells, they may obviate a need for use of additional vascular cell types [23] or different gene manipulation strategies [24] to facilitate vascularization of the newly formed cardiac muscle.

One of the major concerns in using ESCs or their differentiated progeny for cell-based interventions is the risk of neoplastic tumor or teratoma [10] formation due to undifferentiated ESCs. Recent reports describe extensive teratoma formation following injection of ESCs [25], [26]. In one such study, the authors reported tumor formation in 21% of the mice [26] receiving cells. We have previously demonstrated that no expression of *Nanog* or *Pou5f1* was detected following *Nkx2-5^(+)^* CPC derivation and extended culture *in vitro*. In contrast, colonies of undifferentiated ESCs and high expression levels of *Nanog* and *Pou5f1* were found in cultures of the sorted GFP^(−)^ cells [14]. Importantly, in this study we observed no evidence of neoplastic cell growth in the mouse hearts as long as 1 month after cell injections. While these results, along with similar findings in our previous study [14], strongly suggest that the narrow differentiation potential of CPCs could be safe for cell-based therapies, longer grafting times must be studied to further validate these conclusions.

A major drawback of any allogeneic transplantation therapy is the immune response which leads to the rejection of transplanted tissue. In our study we successfully utilized animals with common genetic background to the donor cells [27] trying to avoid immunorejection or the need for immunosuppressive therapy. Even though it has been argued that ESCs and their differentiated progeny are capable of evading immunorejection [7], two reports have demonstrated large-scale host immune response against transplanted ESCs [10], [28]. In a clinical setting this issue must be addressed. Recent studies have proposed several alternatives including somatic cell nuclear transfer [29], and induced pluripotent stem cells [30].

The success of any cell-based therapy for myocardial infarction injury is ultimately judged by the improvement of the heart function, i.e., decreased heart dilatation and increased cardiac contractility and output. Based on the optical mapping of electrical propagation in co-cultures of CPCs and primary ventricular cardiomyocytes, the observed improvement in cardiac mechanical indices in treated hearts could be attributed in part to the functional integration of CPC-derived cardiomyocytes with the host cells. Of note, however, is the relatively low velocity of impulse propagation measured within the CPC islands *in vitro.* This low conduction velocity was consistent with observed sparse Connexin 43 expression and possibly contributed by immature (or mixed from nodal, atrial, and ventricular cells) electrophysiological phenotype of CPC-derived cardiomyocytes [31]. Conceivably, more efficient cardiogenic differentiation within the environment of infracted heart may have yielded higher Connexin 43 expression and maturation of ion currents in engrafted cardiomyocytes resulting in improved functional *in vivo* integration. In support, although no systematic studies were done to assess the arrhythmogenic risk of implanted cells, we observed no difference in the incidence of sudden cardiac death between cell-treated and untreated mice. Moreover, we demonstrate *in vivo* electrical coupling through gap junction formation of the differentiated CPCs with the host myocardium.

It is interesting that CPCs plated at high density within neonatal rat ventricular myocytes primarily differentiated into the cardiomyocyte lineage, while abundant smooth muscle cells were found within the sparsely plated islands [32]. On the other hand, CPCs transplanted in the infarcted hearts primarily differentiated into cardiomyocytes, indicating that the stiffness of the cell attachment substrate [33], density of injected cells, and/or other differences between *in vivo* and *in vitro* microenvironments may all significantly affect their lineage commitment. The systematic studies to understand the factors that control lineage commitment are currently underway.

We detected a number of *βGal* ^(+)^ cardiomyocytes (α-Actinin^(+)^ or MYH6^(+)^) in the host myocardium suggesting the direct differentiation of the delivered CPCs towards the cardiac muscle lineage. Admittedly there is a possibility that some of the detected differentiation events are the result of cell fusion between the host cardiomyocytes and the CPCs. Recently *Kolossov et al*. examined the capacity of both bone marrow derived cells and embryonic stem cell derived cardiomyocytes to engraft into the infracted mouse myocardium [34]. Following bone marrow cell transplantation they detected rare fusion events of the delivered cells with host cardiomyocytes. On the other hand the mouse embryonic stem cell-derived cardiomyocytes colocalized in clusters and could clearly be distinguished from the native cardiomyocytes as a result of their reduced size, distinct cell shape, and incomplete myofibrils, which they concluded was strong evidence that the detected cells were not the result of fusion. Similarly, for the purpose of our study during an early point analysis of the injected myocardium (day 3 post infraction and cell transplantation, data not shown) we detected areas with clustered GFP^(+)^ cells. Although these cells expressed cardiac markers as determined by immunocytochemistry they were small in size and resembled immature cardiomyocytes. Most importantly, one month, post injection 96% of *βGal* ^(+)^ cells contained a single nucleus suggesting their direct differentiation into cardiomyocytes versus their fusion with the host cells.

Importantly, through the systematic assessment of cardiac contractile function, we demonstrated a superior systolic and diastolic performance of the CPC-treated relative to untreated hearts. In particular, while the function of untreated hearts was significantly compromised as early as one week after the infarction (based on echocardiographic parameters), the functional indices in CPC-treated hearts remained relatively constant. The preserved cardiac function was independently confirmed at 4 weeks post-infarction using pressure-volume analysis. Mechanistically, the cardiac function was maintained not only due to attenuated ventricular remodeling including a decrease of scar size and relatively preserved ventricular volume but more importantly due to independently preserved contractile function as evidenced from a higher PRSW slope (a volume independent factor in evaluation of cardiac function in both systole and diastole).

These findings are consistent with previous reports in demonstrating that transplantation of ESC-derived cells leads to the *in vivo* cardiac improvement following myocardial infarction [35], [36]. It is important to note, however, that the mechanisms mediating this effect are currently not known [37]. We acknowledge certain limitations in our work which we plan to further investigate in the future. In particular we are interested in addressing the therapeutic mechanism of the CPCs. More specifically in addition to their direct differentiation capacity into cardiomyocytes, and vascular cells, the CPCs may induce a cardioprotective paracrine effect which in turn induces the host myocardium to regenerate. A genomic expression analysis performed on the CPCs and reported in our previous work indicated that *Vegf, Igf1,* and *Notch* signaling pathways are active in the cells both of which have been shown to have pro-vascular and cardioprotective effects [38], [39]. Moreover, in order to be able to assay *in vivo* functional integration of the donor cells with the host myocardium we plan to utilize genetic calcium indicators and two-photon laser scanning microscopy [40], [41].

An additional important determinant of a successful long-term cell-based cardiac therapy involves the level of cell survival following direct intramyocardial delivery. Previous studies have demonstrated a clear correlation between graft size and the level of functional improvement [42], [43]. Thus cell survival determines graft size and ultimately the level of the therapeutic effect. In general cell survival depends primarily on the initial number of cells delivered, the local cell donor density per injection point, and the status of the tissue in which the cells are being injected (ischemic/inflamed versus normal) [44]. For this study the cell survival capacity of the transplanted CPCs was determined to be 12.7±2.1% which is similar to previous reports utilizing a similar injury and which determined the cell survival to be between 5–9% up to a month following initial cell injections [45], [46] [47]. Finally, in order to maximize the therapeutic effect of the delivered CPCs through increased cell survival it may be necessary to modify the delivery method in order to ensure decreased apoptosis and localized inflammatory response as well as prime the cells to cope with the increased stress associated with the entire process [36].

The recent identification and characterization of adult [48] and ESC-derived CPCs with multipotential but cardiac tissue-restricted differentiation capacity has reinvigorated the field of cell-based cardiac therapies. Taken together, our results clearly demonstrate the therapeutic value of the ESC-derived CPCs in the infarcted mouse heart. While potential strategies to control differentiation fate of CPCs towards blood vessels or cardiac muscle upon implantation remain to be explored, this study establishes the foundation and the rationale for the future testing of human embryonic or induced pluripotent stem cell-derived CPCs for repair of cardiac infarction.

## Materials and Methods

### Cell culture and differentiation

Mouse ESCs (D3 [15] and Rosa26 [16] lines) were cultured and differentiated as previously described [14]. Further detail is provided in **File S1**.

### DNA plasmids and cell transfection

The previously described [22] cardiac specific enhancer element (fragment 8) of the mouse Nkx2-5 transcription factor along with the Hsp68 minimal promoter [49] were excised from the provided plasmid (XhoI/NcoI) and inserted upstream of the enhanced GFP gene in the Bluescript vector. This in conjunction with a plasmid DNA containing the polymerase II promoter at the 5′ end the hygromycin phosphotransferase gene were used to stably transfect mouse ESCs in the presence of hygromycin. A stable clone from each cell line was selected for any further experiments.

### *In vitro* CPC differentiation capacity

To assay their differentiation capacity, sorted GFP^(+)^ CPCs were re-aggregated for 24 hours in suspension and re-plated on fibronectin coated tissue culture dishes and further maintained for seven days in ESC differentiation medium.

### Quantitative RT-PCR

Embryoid bodies cultured in hanging droplets or in suspension were harvested every 24 hours in order to isolate total RNA. For the quantitative RT-PCR analysis total RNA (2 µg) was reverse transcribed using random decamers as primers (Ambion, cat.# 57226), and the MMLV-RT enzyme (Invitrogen, 28025-013). Relative RNA expression levels were measured using quantitative RTPCR. Detection of the described markers was achieved with FAM/MGB probes (Applied Biosystems): Nkx2.5 (Mm00657783_m1), Myh6 (Mm00440354_m1), Brachyury (Mm00436877_m1). The instrument used was the Applied Biosystems ABI Prism 7900HT sequence detection system and the software for data collection and analysis was the SDS2.1. An 18S RNA probe (Hs99999901_s1) was used in order to normalize the data.

### Co-culture of CPCs and NRVMs

Fibronectin-coated coverslips and polydimethylsiloxane (PDMS) rings were used to selectively plate isolated CPCs only in the central region (5 mm diameter) of the coverslips (low density: 5×10^4^ cells, high density: 2.5×10^5^ cells). Following CPC attachment floating cells were washed off and the PDMS ring was removed. Isolated NRVMs were subsequently plated around the CPCs. Washing of unattached NRVMs within 6–8 hours following plating ensured minimum NRVM attachment on top of the CPCs.

### Optical mapping of membrane potentials and immunostaining in CPC-NRVM cocultures

Electrical propagation in CPC/NRVM cocultures was optically mapped using a bundle of 504 hexagonally arranged optical fibers (RedShirtImaging) providing a spatial resolution of 750 µm in a 19.5 mm field of view, as previously described [18], [50]. Briefly, the cocultures were incubated for five minutes with a voltage sensitive dye (di-4 ANEPPS, 16 µM) at room temperature, placed in a heated recording chamber, perfused with Tyrode's solution, and illuminated with a green excitation light (520±30 nm). The recorded red fluorescence (>590 nm) was transferred through the fiber optic bundle, converted to voltage using photodiodes, amplified, sampled at 2.4 kHz, and stored on a PC. Before the onset of pacing, all cocultures were checked for the presence of spontaneous activity. A XYZ-micropositioned bipolar platinum electrode was then used to locally stimulate the CPC area in the center of the monolayer and propagated action potentials were optically mapped. Electrical stimulation, light exposure, and data acquisition were synchronized using custom LabView software. Data analysis was performed using MATLAB as previously described [18], [51]. Activation times were defined as times of 50% upstroke of the action potential and used to construct isochrone maps. Local conduction velocities were calculated using the activation time for each optical channel relative to those of neighboring channels[18].

Following optical mapping analysis the samples were fixed in 4% parafolmaldehyde and stained with antibodies against the GFP protein (Molecular probes, A21311, A11120), α-Actinin (Sigma, A7811), Acta2 (Abcam, ab5694), and Connexin 43 (Sigma, C6219). Secondary antibodies were purchased from Molecular probes and were conjugates to the fluorochromes Alexa 488, and Alexa 546. Nuclei were stained with 4′,6-diamidino-2-phenylindole (Molecular probes). The samples were imaged with a Nikon TE2000 and a confocal Zeiss 510 microscope.

### Animal Model

All research involving experimentation on vertebrate animals have been specifically approved by Johns Hopkins Medical Institutions (Animal Protocol # MO0M296). Using a left anterior thoracotomy, the heart of SVE129 female mice (8–10 weeks of age) was exposed and LAD was ligated permanently. For sham surgeries, the needle was passed under the LAD without ligation. The mice were monitored twice a day for four weeks. Four experimental groups were established consisting of animals that underwent LAD ligation and received phosphate buffered saline injections with (n = 15) or without cells (n = 10) and animals that underwent sham surgeries and also received saline injections with (n = 7) or without cells (n = 11). Immediately following infarction injury, each animal received three injections (10 µl/injection) of saline or saline with CPCs (0.5–1×10^6^ cells total) in the boundaries and the center of the infarcted region. The cells used in these experiments were GFP^+^ CPCs isolated on differentiation day 6. The animals were sacrificed four weeks post-transplantation and the hearts prepared for immunohistological analysis.

### CPC injections

Using a tubing system attached to a standing syringe, we injected 4–5×10^5^ cells in three doses (10 µl/injection) directly into myocardium with a 30-gauge needle. Injections were done immediately following LAD ligation, into the mid infarct zone and border-zones. The infarct zone was defined by blanching of the heart after ligation.

### Echocardiography

The analysis was done using a HP/PHILIPS/AGILENT SONOS 5500. The animals were analyzed prior to surgery (same day) and at 1, 2, and 4 weeks after it. The procedures were performed under general anesthesia using isoflurane 1–2%.

### Pressure volume loop analysis

4 weeks after the surgery the mice were anesthetized using a combination of etomidate, morphine and urethane, which were injected intraperitoneally. Using the right carotid approach a Millar SPS 839 catheter progressed to the left ventricle. During the procedure, a diluted 6.25% albumin solution was infused into jugular vein at a rate of 5 mcl/min. Pressure volume data were obtained at baseline with closed chest and after opening the thorax with IVC occlusion. The volume calibration was done using thoracic aortic flow and hypertonic saline infusion.

### Statistical analysis

We obtained average and standard error of mean for all values and used one way and two way ANOVAs for statistical analysis (*Graph-pad prism 4.03*)**.**

## Supporting Information

File S1 Cell culture and differentiation, FACS analysis and sorting, isolation of nrvms, harvesting the hearts and tissue preparation, immunocytochemistry, statistical analysis.
(0.06 MB DOC)

Figure S1 Cardiomyocyte and smooth muscle lineage committed mouse ESC-derived CPCs functionally electrocouple with neonatal rat ventricular cardiomyocytes. (a–b) *In vitro* co-culture of ESC-derived CPCs with NRVMs. The CPCs were selectively plated at low density in the center region of the coverslip and subsequently differentiated into cardiomyocytes and smooth muscle cells. A confluent layer of NRVMs surrounded the CPCs. Dashed lines denote the border between the two cell types. Mouse ESC-derived cardiomyocytes as well as NRVMs stain positive for α-Actinin (red), whereas only mouse ESC-derived CPCs and mouse ESC-derived cardiomyocytes express GFP (green). (c–d) High magnification immunostaining analysis illustrates the interface of the two cell types demonstrating formation of Connexin 43 gap junctions. (e) The majority of CPCs plated at low density differentiated into smooth muscle cells (e, green) with some cardiomyocyte differentiation (e, red). (f) Sequential isopotential maps reveal electrical propagation from the central island of differentiated CPCs to the outside regions containing primary cardiomyocytes (central island is indicated by dashed white circle). Color bar shows relative transmembrane potential normalized from resting (blue) to peak (red) value. The monolayer was electrically stimulated by a point electrode in the center, indicated by the yellow pulse sign in 0 ms frame. The timing index has been set relative to the stimulus pulse (indicated by the yellow triangle on membrane potential traces). Membrane potential traces are from three channels (channel 1 in the central island of cardiac progenitor cells and channels 2 and 3 in the outside region with cardiac cells). Note that electrical propagation was very slow inside the central island due to predominance of CPC-derived smooth muscle cells and significantly accelerated after exiting to the outside cardiomyocyte region.
(3.21 MB TIF)

Figure S2 Kaplan-Meyer survival graph showing the four study arms during the four weeks after myocardial infarction. Logrank analysis for survival demonstrated no significance in the number of animal deaths in the four experimental groups.
(1.25 MB TIF)
