# Supplementary material for: Implantation of Mouse Embryonic Stem Cell-Derived Cardiac Progenitor Cells Preserves Function of Infarcted Murine Hearts
Source: PLoS One. 2010 Jul 12;5(7):e11536. doi: 10.1371/journal.pone.0011536 (PMC2902505; doi:10.1371/journal.pone.0011536)
Supplement: File S1 — Cell culture and differentiation, FACS analysis and sorting, isolation of nrvms, harvesting the hearts and tissue preparation, immunocytochemistry, statistical analysis. (0.06 MB DOC) [file pone.0011536.s001.doc]

**Supplementary Material**

**Experimental Techniques.**

**Cell culture and differentiation**. Mouse ESCs (D3 and Rosa26 lines) were cultured and passaged on supporting feeder layers of mitotically inactivated primary mouse embryonic fibroblasts in ESC medium consisting of: DMEM, 10% fetal bovine serum (Catalog # S11550, Lot # A0036, Atlanta Biologicals), Glutamax-1, Non-essential amino acids, sodium pyruvate, β-mercaptoethanol, Gentamycin, and ESGRO leukemia inhibitory factor (LIF, Chemicon). To induce differentiation, following feeder layer subtraction, cells were resuspended in ESC medium (5x10^4^ cells/ml) in the absence of ESGRO LIF. Differentiation through embryoid body (EB) formation was induced using the hanging droplet technique with each drop containing approximately 10^3^ cells (20 μl/drop). Two days following initiation of differentiation the EBs were transferred in suspension on poly-HEMA-treated tissue culture dishes in order to avoid cell attachment. At this time point the mouse ESC medium was supplemented with ascorbic acid (0.1mg/ml). The EBs were cultured in suspension until differentiation day 6 at which point the cells were enzymatically dissociated (0.25% Trypsin/EDTA) and the GFP^+^ cells were selectively isolated using fluorescence activated cell sorting (FACS Aria).

**FACS analysis and sorting**. In order to determine the yield of ESC-derived GFP^+^ CPCs the stably transfected ESCs were differentiated using the hanging droplet technique and EBs were harvested at the indicated time points (differentiation days 6-10). Each sample contained approximately 100 EBs. The percentage of GFP^+^ cells was determined in a FACS Calibur system (BD Biosciences). Age-matched untransfected mouse ESCs were used as a negative control.

In order to isolate the mouse ESC-derived GFP^+^ CPCs we utilized the FACSAria sorting system (BD Biosciences). EBs were dissociated on differentiation day 6 and resuspended in a low FBS (1%) containing medium. Following cell sorting the GFP^+^ cells were either cultured further in order to analyze their *in vitro* differentiation capacity or kept briefly at 4^o^C prior to injections into the myocardium.

**Isolation of neonatal rat ventricular myocytes (NRVMs).** Cardiac cells were dissociated from the ventricles of 2 day old neonatal Sprague-Dawley rats (Harlan) using Trypsin (US Biologicals) and Collagenase (Worthington) and resuspended in culture medium supplemented with 10% calf serum and 10% horse serum, as previously described [1]. The purity of the cardiomyocyte cell culture (>93%) was confirmed by immunocytochemistry (sarcomeric α-actinin, data not shown). The isolated cells were plated at density of 2.5x10^5^ cells/cm^2^ on fibronectin-coated coverslips (Aclar®, Electron Microscopy Sciences) following initial plating of the CPCs. After 2 days of culture, the media was changed to M199 (Invitrogen) supplemented with 2% FBS, 1% HEPES, nonessential amino acids, glucose, L-glutamine, vitamin B12, and penicillin G [1].

**Harvesting the heart and tissue preparation**. Immediately after finishing the pressure-volume recording, the hearts were harvested. After initial perfusion with potassium chloride (20mM) for 10 minutes, we used a 4% parafolmaldehyde solution to perfuse the hearts, for another 10 minutes through aorta. The hearts were kept overnight in 4% PFA, transferred to a glycine solution, and then incubated overnight in 30% sucrose for dehydration. The hearts were sectioned at fixed intervals and the thick tissue sections were either cryopreserved in OCT (optimal cryogenic temperature, Tissue Tek, Miles, Naperville, IL) at –80^o^C or embedded in paraffin and kept at 4^o^C.

In order to detect the level of scarring and cell engraftment into the host myocardium, parallel thin sections of the paraffin preparations were stained either using Mason’s trichrome dye or with an antibody against β-Galactosidase (Abcam, ab616) which is constitutively expressed in the Rosa26 cells.

**Immunocytochemistry**. For the purposes of the *in vitro* characterization of the differentiating CPCs, following cell fixation with 4% parafolmaldehyde, the cell membrane was permeabilized using a saline solution containing 0.02% Triton X-100. Prior to primary antibody addition the cells were blocked with a saline solution containing 10% Goat serum. A similar technique for sample preparation was used for the cryosectioned mouse hearts. Antibodies used: Mouse anti α-Actinin (IgG1, Sigma, A7811), Rabbit anti Smooth Muscle Actin (IgG, Abcam, ab5694), Rabbit anti Von Willebrand factor (IgG, Abcam, ab6994), Rabbit anti GFP (IgG, Invitrogen, A11122), Rabbit anti GFP – Alexa 488 conjugated (IgG, Invitrogen, A21311), Rabbit anti Connexin 43 (IgG, Sigma, C6219), Mouse anti Myosin Heavy Chain (IgM, Abcam, ab24642), Mouse anti N-Cadherin (IgG1, Affinity Bioreagents, MA1-2002), Rabbit anti KDR (IgG, Abcam, Ab2349), Mouse anti β-Galactosidase (IgM, Sigma, G6282), Mouse anti β-Galactosidase (IgG_2b_, Zymed, 03-2100), Goat anti Mouse IgG – Alexa 488 (Invitrogen, A11029), Goat anti Mouse IgM – Alexa 488 (Invitrogen, A21042), Goat anti Rabbit – Alexa 488 (Invitrogen, A11008), Goat anti Mouse IgM – Alexa 594 (Invitrogen, A21044), Goat anti Mouse IgG – Alexa 594 (Invitrogen, A11032). β-Actin was detected with Alexa fluorochrome-conjugated Phalloidin (Invitrogen). Nuclei were detected with 4',6-diamidino-2-phenylindole (DAPI, Invitrogen, D3571).

**Statistical Analysis**. We used the echocardiographic data from individual animals and ran one way ANOVA tests with repeated measures to find the differences within each animal.  Since we already had done two-way ANOVAs to compare treatment vs. control, we decided not to repeat it.

Running one way ANOVA with repeated measures to account for several measurements within each animal for echocardiographic evaluation of ejection fraction (EF), fractional shortening (FS), left ventricular size in diastole (LVIDd) and systole (LVIDs) showed no significant change over four weeks of follow up in each sham group and also CPC treated infarction arm. However mice with MI within control group showed significant change over time supporting decreased contractility and increased ventricular size. Following are the P values for the analysis. The last column is the number of mice we performed the statistical analysis on within each group.

|  | LVIDd | LVIDs | FS | EF | N |
| --- | --- | --- | --- | --- | --- |
| MI-CPC | 0.0545 | 0.0626 | 0.0762 | 0.1312 | 8 |
| MI-Saline | 0.042 | 0.025 | 0.027 | 0.012 | 3 |
| Sham-CPC | 0.631 | 0.6152 | 0.8005 | 0.3985 | 9 |
| Sham-Saline | 0.2303 | 0.5944 | 0.1109 | 0.6623 | 4 |

1. Bursac N, Parker KK, Iravanian S, Tung L (2002) Cardiomyocyte cultures with controlled macroscopic anisotropy: a model for functional electrophysiological studies of cardiac muscle. Circ Res 91: e45-54.
