# Supplementary figures and images for: Implantation of Mouse Embryonic Stem Cell-Derived Cardiac Progenitor Cells Preserves Function of Infarcted Murine Hearts
Source: PLoS One. 2010 Jul 12;5(7):e11536. doi: 10.1371/journal.pone.0011536 (PMC2902505; doi:10.1371/journal.pone.0011536)

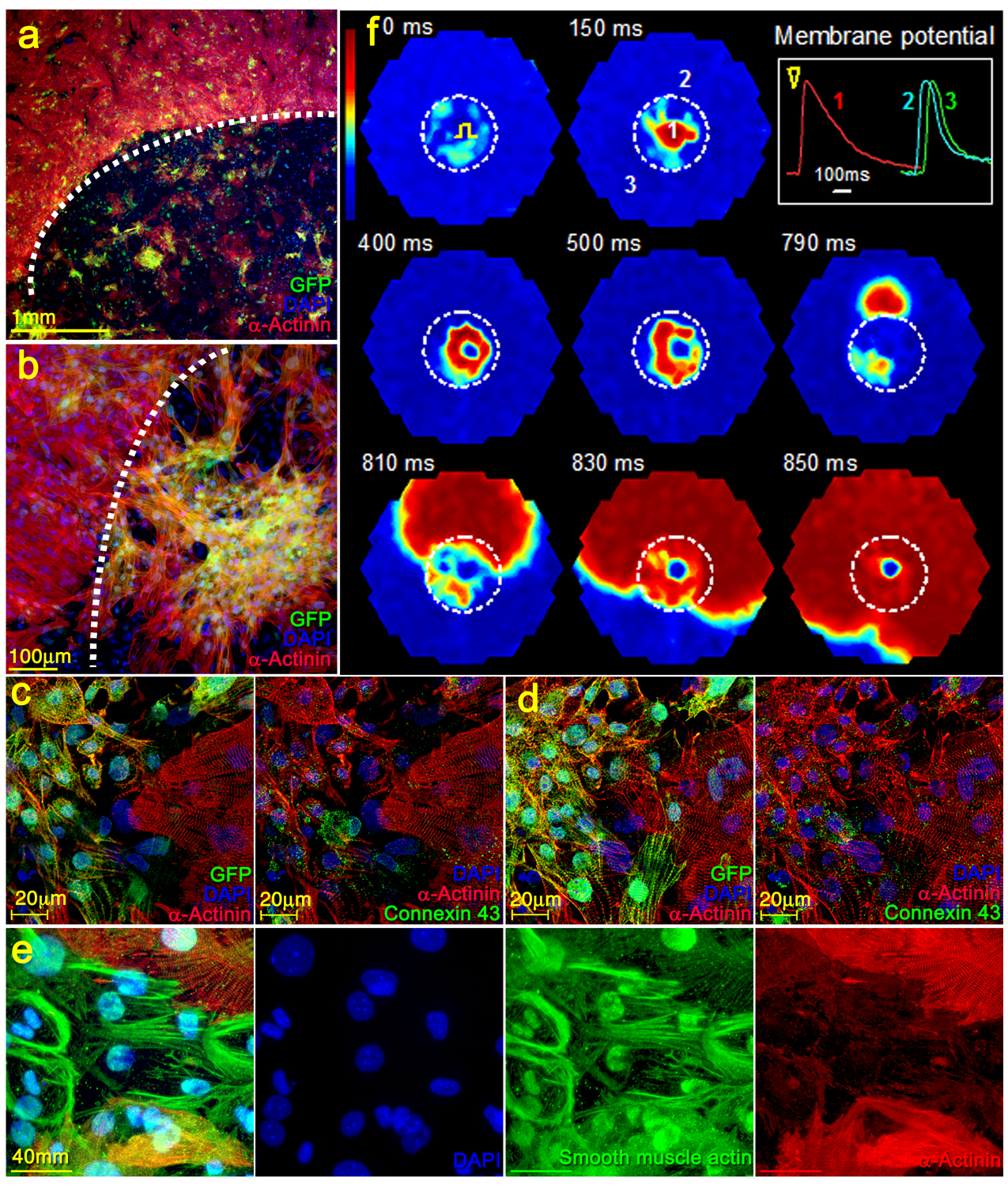

Supplement: Figure S1 — Cardiomyocyte and smooth muscle lineage committed mouse ESC-derived CPCs functionally electrocouple with neonatal rat ventricular cardiomyocytes. (a–b) In vitro co-culture of ESC-derived CPCs with NRVMs. The CPCs were selectively plated at low density in the center region of the coverslip and subsequently differentiated into cardiomyocytes and smooth muscle cells. A confluent layer of NRVMs surrounded the CPCs. Dashed lines denote the border between the two cell types. Mouse ESC-derived cardiomyocytes as well as NRVMs stain positive for α-Actinin (red), whereas only mouse ESC-derived CPCs and mouse ESC-derived cardiomyocytes express GFP (green). (c–d) High magnification immunostaining analysis illustrates the interface of the two cell types demonstrating formation of Connexin 43 gap junctions. (e) The majority of CPCs plated at low density differentiated into smooth muscle cells (e, green) with some cardiomyocyte differentiation (e, red). (f) Sequential isopotential maps reveal electrical propagation from the central island of differentiated CPCs to the outside regions containing primary cardiomyocytes (central island is indicated by dashed white circle). Color bar shows relative transmembrane potential normalized from resting (blue) to peak (red) value. The monolayer was electrically stimulated by a point electrode in the center, indicated by the yellow pulse sign in 0 ms frame. The timing index has been set relative to the stimulus pulse (indicated by the yellow triangle on membrane potential traces). Membrane potential traces are from three channels (channel 1 in the central island of cardiac progenitor cells and channels 2 and 3 in the outside region with cardiac cells). Note that electrical propagation was very slow inside the central island due to predominance of CPC-derived smooth muscle cells and significantly accelerated after exiting to the outside cardiomyocyte region. (3.21 MB TIF) [file pone.0011536.s002.tif]

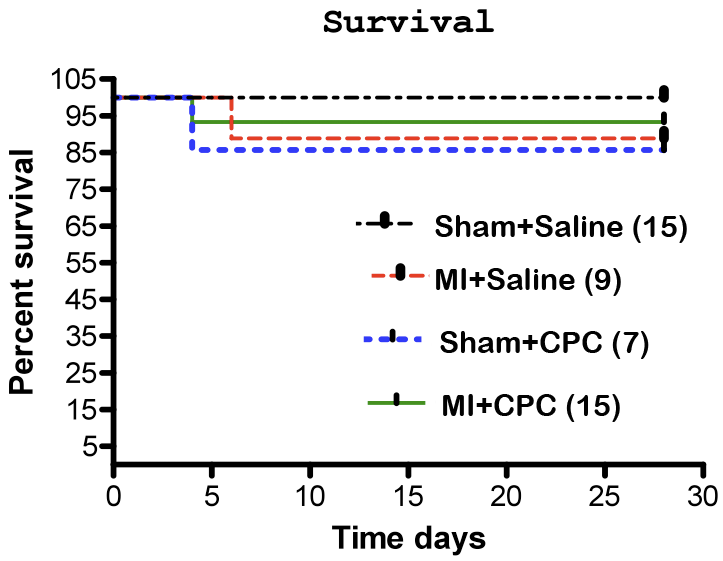

Supplement: Figure S2 — Kaplan-Meyer survival graph showing the four study arms during the four weeks after myocardial infarction. Logrank analysis for survival demonstrated no significance in the number of animal deaths in the four experimental groups. (1.25 MB TIF) [file pone.0011536.s003.tif]
